# Spatiotemporal Difference Characteristics and Influencing Factors of Tourism Urbanization in China’s Major Tourist Cities

**DOI:** 10.3390/ijerph181910414

**Published:** 2021-10-03

**Authors:** Xia Xie, Lei Zhang, Hui Sun, Feifei Chen, Chunshan Zhou

**Affiliations:** 1 Key Laboratory for Sustainable Development of Xinjiang’s Historical and Cultural Tourism, Xinjiang University, Urumqi 830046, China; xiexia@xju.edu.cn (X.X.); zhanglei@stu.xju.edu.cn (L.Z.); sunhui920@stu.xju.edu.cn (H.S.); chenfei@stu.xju.edu.cn (F.C.); 2 College of Tourism, Xinjiang University, Urumqi 830046, China; 3 School of Geography and Planning, Sun Yat-Sen University, Guangzhou 510275, China

**Keywords:** tourism urbanization, state space method, panel data model, major tourist cities in China

## Abstract

Tourism is crucial for promoting industrial development and is an important driver of China’s new type of urbanization. A tourism urbanization index system was constructed in three dimensions: the tourism industry, urbanization, and the ecological environment. The spatial–temporal differentiation characteristics and influencing factors of tourism urbanization in 35 major tourist cities in China from 2009 to 2018 were analyzed using the state space method, standard deviation ellipse, and spatial autocorrelation analysis. The results show the following. (1) Over time, the tourism industry index displays an upward trend, the urbanization index exhibits a more obvious upward trend, and the ecological environment index fluctuates strongly. Under the influence of all three factors, the tourism urbanization index shows a fluctuating rising trend. (2) Regarding the spatial distribution pattern, the development center of tourism urbanization shifts to the southeast, and the distribution direction is northeast-southwest. There is a significant agglomeration in global spatial autocorrelation. The local spatial correlation pattern is dominated by correlation characteristics and supplemented by different characteristics. (3) In terms of influencing factors, policy and regional development strategy, tourism resource endowment, economic development level, and traffic conditions are listed in descending order of influencing degree. Finally, we put forward some suggestions.

## 1. Introduction

Over the past 40 years, China’s urbanization has undergone major changes and made great progress. China’s urbanization rate grew from 17.92% at the beginning of the reform and opening up to 60.60% in 2019. In 2019, the tourism industry accounted for 11.05% of the GDP, which illustrates its strength as a driving force [1]. The progress of urbanization has entered a crucial stage. Therefore, simply pursuing the speed of urbanization cannot meet the needs of society and people’s living standards. Only through policy guidance to drive the consumer market can the enthusiasm and creativity of various departments and related enterprises be stimulated. In the new era, China’s goal for its ecological environment is sustainable development. On this basis, promoting the development of tourism, vigorously pursuing urbanization and improving the ecological environment are important measures for China as the nation faces new patterns of regional development and sustainable economic development [2]. How to accomplish common development of the tourism industry and urbanization within the context of the ecological environment in order to maximize tourism advantages and optimize urbanization construction has emerged as a pressing issue.

China has witnessed a rapid increase in urbanization. The 18th National Congress of the Communist Party of China further emphasized the importance of urbanization. “New urbanization” has received great attention from all parties. It has become a key force in China’s economic growth and social development [3]. As a comprehensive industry, tourism involves multiple departments and industries. Therefore, in the current wave of urbanization, the tourism industry has become a new impetus in the development of urbanization. In China’s new period, tourism urbanization is the product of social development. This has provided a new direction to explore in the development of urbanization. Cities are not only the spatial carriers of human survival and social development but also spatial places for economic, political, and cultural agglomeration. Therefore, the quality of the urban ecological environment quality is a crucial consideration for tourists [4]. However, while tourism urbanization brings economic and social benefits, it can also damage the local ecological environment. The excessive development of tourism resources can lead to the destruction of the ecological environment; the booming development of the tourism industry inevitably brings about the flow of the urban population, which leads to environmental “overload” and other problems. Strengthening ecological civilization development is essential in improving the quality and efficiency of development in a new stage of development. The report of the 18th National Congress of the Communist Party of China framed the construction of an ecological civilization as a central concern. After five years, the report of the 19th National Congress of the Communist Party of China again proposed acceleration of the construction of ecological civilization, emphasizing the harmonious development of humans and nature [5]. Therefore, the ecological environment is not only the foundation for tourism, but also a necessary condition for urbanization.

Through the above analysis, to avoid the one-sided view of tourism urbanization as the interaction between the tourism industry and urbanization, the ecological environment problems in the process of tourism urbanization should not be ignored. The tourism urbanization system should be regarded as an organic whole formed by the interaction of the three subsystems of the tourism industry, urbanization, and the ecological environment [6,7]. An in-depth analysis of the space-time distribution features of the three subsystems can help us better understand the development state and trend of tourism urbanization. First, the tourism industry and urbanization interact. The development of the tourism industry can bring significant economic benefits to cities and improve urban infrastructure construction. Moreover, the expansion of urbanization benefits the tourism industry in terms of people, transportation, and communication [8]. Second, the tourism industry and ecological environment influence each other. The development of the tourism industry relies on the development of resources and ecology. A good ecological environment provides a solid foundation for the tourism industry. However, the excessive development of the tourism industry inevitably has adverse consequences for the ecological environment [9]. Finally, the interaction between urbanization and the ecological environment has both beneficial and negative effects. Reasonable and scientific urban planning improves resource utilization efficiency and promotes the development of the ecological environment. In contrast, unreasonable urbanization damages the ecological environment. Similarly, the ecological environment provides a fundamental guarantee for the development of urbanization and optimizes the industrial structure in the region [10]. The tourism industry is a dominant driving force of urbanization and the ecological environment, urbanization is an important foundation, and the ecological environment is the material carrier. There is still a lack of research on the interaction among these three factors. Existing studies mainly focus on the interactions between them, such as the tourism industry and urbanization [11,12,13], urbanization and the ecological environment [14,15,16], and the ecological environment and the tourism industry [17,18,19].

Therefore, this paper takes 35 major tourist cities in China as its research objects, and constructs a tourism urbanization index system with three subsystems: the tourism industry, urbanization, and the ecological environment. The state space method and spatial autocorrelation analysis are used in this research to investigate the properties of time change and spatial difference. Combined with a panel data model, the influencing factors of spatial–temporal differentiation characteristics are analyzed. It is conducive to grasping the spatial and temporal differentiation characteristics of the comprehensive development level of the tourism industry, urbanization and the ecological environment, and understanding the impact of various factors on tourism urbanization. It provides a theoretical basis for China to vigorously develop the tourism industry and build on new urbanization and sustainable development plans within the ecological environment. Furthermore, it helps to further adjust China’s tourism industrial structure, accelerate the urbanization process, and build a beautiful China.

## 2. Literature Review

The research on tourism urbanization originated in the late 20th century. Mullins first proposed that tourism urbanization is a type of urbanization formed through the rapid development of economic restructuring and changes in urban space [20]. Subsequently, different scholars have put forth different definitions of the concept of urbanization [21]. From the perspective of consumption, Lu and Ge pointed out that tourism urbanization is the change in people’s urbanization process from traditional consumption to pleasure consumption [22]. An proposed that tourism urbanization is a process of promoting economic and social transformation as well as cultural reconstruction through the tourism industry, thereby promoting urbanization development [23]. In recent years, scholars have gradually increased their research on the relationship among the tourism industry, urbanization, and the ecological environment. Burak studied the impact of tourism urbanization on Turkey’s ecological environment. The local government’s measures to encourage tourism investment have accelerated the development of urbanization but have also brought about ecological and environmental problems, such as the salinization of seawater and the loss of farmland [24]. On the basis of analyzing tourism satellites, Jones and Munday proposed that, while developing tourism urbanization, we must attach importance to the protection and management of the ecological environment and adhere to the ecological concept of sustainable development [25]. Zhang and Li [26], Hu et al. [27], and Yang et al. [28] studied Heilongjiang, Poyang Lake, and Ningxia, respectively, in China. Studies show that the development of urbanization and the tourism industry are on the rise. There are considerable variances in the rate of increase between regions. The development trend of the ecological environment presents an overall increase and a partial decrease. The development level of the ecological environment is higher than that of urbanization and the tourism industry. The development of tourism urbanization in China has obvious spatial agglomeration characteristics. The global and local spatial autocorrelation is significant.

For the study of spatiotemporal evolution characteristics of tourism urbanization, Deng [29] took the cities of Shanxi Province as the research object; calculated the comprehensive index of urbanization, tourism industry, and ecological environment; and pointed out that the influencing degree of each system was different for each prefecture-level city. Ma and Lv [30], Liu [31], and Liu et al. [32] studied the spatial and temporal evolution process of Zhangjiajie, Wuling Mountain Area in Hunan and 12 provinces in the western region through the response intensity of tourism urbanization. Xiong et al. [7], Rong et al. [33], and Han et al. [34] conducted empirical analyses on the tourism industry, urbanization, and the ecological environment in the Dongting Lake region, southern Anhui Province, and the Silk Road Economic Belt, respectively. It can be seen that the development of tourism urbanization cannot be simply attributed to the interaction between the tourism industry and urbanization. In regions with a developed tourism industry, more attention should be given to the protection of the ecological environment.

Analysis of the driving factors of tourism urbanization is the key to achieving sustainable development, which can further guide the formulation and planning of relevant policies. Scholars have studied the influencing factors of tourism urbanization in many dimensions. By comparison, we can find that policy factors are particularly prominent in the Chinese context. In addition, tourism urbanization is affected by tourism resource endowment, economic development level, transportation facilities, location conditions, and other factors [7,31]. Çevirgen and Kesgin proposed that the driving factor of tourism urbanization in Alania in Turkey is the development of real estate [35]. When analyzing the urbanization of West Bengal, Dandapath and Mondal pointed out that the convenience of transportation has improved the development of the tourism industry [36]. Lu et al. put forward the factors of tourism urbanization development in Tangkou town, including location, policy, resources and residents’ participation [37]. Cheng and Xu proposed that the main factors affecting the spatial–temporal coupling of urbanization, tourism, and ecology in China are geographical location, industrial structure, and openness [38].

In general, the extant research on the relationship among the tourism industry, urbanization, and the ecological environment is still insufficient, as researchers tend to ignore ecological problems in the process of tourism urbanization development. At present, there are more empirical studies than theoretical studies in this area [39,40]. In terms of the research, most studies are concentrated in economically developed regions. There are few articles from the macroscopic perspective. Most of the existing research methods use the entropy weight method and coupling coordination degree model, and few use the state space method. The state space method takes into account the three-dimensional factors of the tourism industry, urbanization, and the ecological environment, and more intuitively shows the inherent relationship of the tourism urbanization system. Scholars’ research on the driving factors of tourism urbanization mainly focuses on capital, resources, economy and transportation, and less on the change processes of driving mechanisms.

With the increase in people’s quality of life, the demand for tourism is gradually increasing. Achieving high-quality tourist growth is a prerequisite of the modern day and a major driving force for societal advancement. Few studies have been conducted on tourism urbanization in tourist cities. Such research represents a vital pathway to accelerate industrial upgrading and improve economic efficiency to clarify the spatial–temporal distribution features and influencing factors of tourism urbanization in different regions. Therefore, analyzing the spatial and temporal differentiation characteristics and influencing factors of tourism urbanization in major tourist cities in China is conducive to promoting the rapid and effective development of the tourism industry and in providing theoretical support and a practical basis for the construction of tourism urbanization in various regions.

## 3. Materials and Methods

### 3.1. Study Area and Data Sources

**Figure 1:**
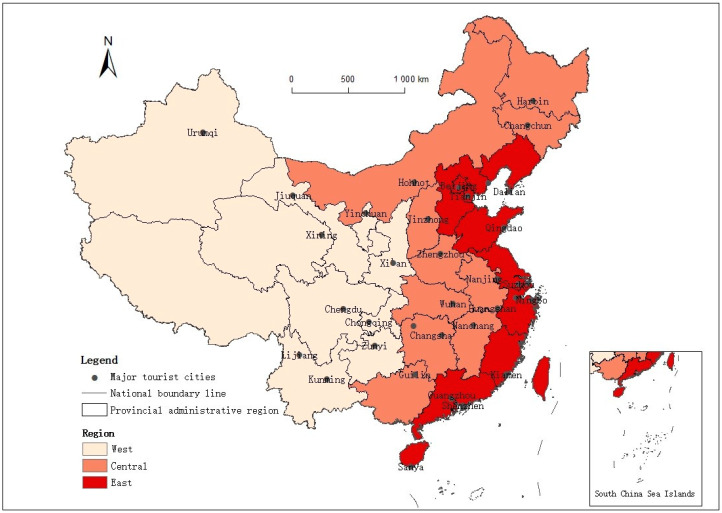
Major tourist cities in China.

The regions studied in this paper are the major tourist cities in China. Referring to the division of tourist cities by relevant scholars [41], the study follows the principles of scientificity and representativeness. Based on the urban function theory of urban geography [42], the tourism development level of cities is measured according to the total amount of tourism reception and the proportion of tourism income in GDP. Given its vast territory, China is divided into three regions: eastern, central, and western. According to the availability of data, 35 major tourist cities were screened (Figure 1). Among them, 14 cities are in east China, 11 cities are in central China, and 10 cities are in west China. These tourist cities have a certain functional scale and status in terms of urban functions. There are four municipalities: Beijing, Tianjin, Shanghai, and Chongqing. Key provincial capitals include Nanjing, Hangzhou, Guangzhou, Zhengzhou, Wuhan, and Changsha. In addition, internationally renowned tourist cities, such as Suzhou, Sanya, Xi’an, and Chengdu, were included.

The panel data of major tourist cities in China from 2009 to 2018 were selected. The data come from the statistical yearbooks of 35 cities, the statistical bulletin of national economic and social development, the “China Tourism Statistical Yearbook”, and the “China City Statistical Yearbook”. For some cities where individual data are difficult to obtain, the weighted average method is used to supplement the values.

### 3.2. Indicators System Construction

At present, scholars have different indexes for the construction of tourism urbanization index system. This paper argues that tourism urbanization means that, through the development of the tourism industry, the region has changed its traditional mode of urbanization development and the quality of the ecological environment, and has promoted the aggregation of population, industry, and the expansion and reconstruction of space. Therefore, tourism urbanization pays more attention to sustainability and relies on the promotion of tourism industry to accelerate the process of urbanization. With reference to the relevant literature [26,27,28,29], this paper constructs the evaluation index system of tourism urbanization from the three dimensions of the tourism industry, urbanization and the ecological environment (Table 1). Among them, the urbanization subsystem only reflects the continuous increase in urban population and the continuous expansion of urban scale. It does not involve the tourism economy and environmental quality. Since social and economic development levels differ across major tourist cities in China, we should consider the relevance, scientificity, and data availability of index selection to comprehensively and objectively reflect the development level of tourism urbanization in each city [43,44,45]. Based on the previous research results [46], this paper adds the number of A-level scenic spots and end-of-year mileage per capita. A-level scenic spot is a comprehensive evaluation of the quality level of scenic spots in China. It is based on tourism resources, taking into account the ecological environment, service quality, and tourist experience and other evaluation factors. To some extent, it reflects the endowment of local tourism resources and the development of the tourism industry. The higher the number of A-level scenic spots, the more they can attract tourists, stimulate tourism economic growth, form industrial agglomerations, and promote the development of tourism urbanization. Per capita mileage at the end of the year represents the traffic conditions and the development of urbanization.

The tourism urbanization evaluation index system of China’s major tourist cities constructed in this paper includes 28 evaluation indexes. Among them, the tourism industry subsystem is composed of tourism flow, tourism benefit, and tourism facilities, which reflects the development level of the tourism industry. The urbanization subsystem is composed of the economy, society, and population, which reflects the development scale of urbanization. The ecological environment subsystem considers the three aspects of level, pressure, and construction, which reflects the protection and development of the ecological environment. In Table 1, “+” is a positive indicator. “−” is a negative indicator. The positive index indicates that the index value is positively correlated with the subsystem, that is, the larger the index value, the higher the evaluation level of the subsystem. The negative index indicates that the index value is negatively correlated with the subsystem, that is, the smaller the index value, the higher the evaluation level of the subsystem.

### 3.3. Methods

#### 3.3.1. Entropy Weight Method

This paper uses the entropy method to evaluate tourism urbanization [47,48]. The specific calculation process is as follows. The results are shown in Table 1:

(1) Using the range method to standardize the original data:
  (1) $$\mathrm{Positive}\;\mathrm{indicators}:\;x^{\prime }\theta ij=(x\theta ij-\min(x\theta ij))/(\max(x\theta ij)-\min(x\theta ij))$$
  (2) $$\mathrm{Negative}\;\mathrm{indicators}:\;x^{\prime }\theta ij=(\max(x\theta ij)-x\theta ij)/(\max(x\theta ij)-\min(x\theta ij))$$
  where *θ* is the year, *i* is the province, *j* is the index, and xθij is the *j*-th index value of province *i* in year *θ*.
(2) The original data may appear as zero after dimensionless analysis. To avoid the meaninglessness of individual indicators in the following calculation, the numerical value after becoming dimensionless is translated:
  (3) $$x^{\prime \prime }\theta ij=x^{\prime }\theta ij+A(A\;\mathrm{is}\;\mathrm{the}\;\mathrm{translation}\;\mathrm{amplitude})$$
(3) Determine the weight of indicators:
  (4) $$p\theta ij=x^{\prime \prime }\theta ij/\sum_{i=1}^{n}x^{\prime \prime }\theta ij(\theta =1,2,\ldots r;i=1,2,\ldots n;j=1,2,\ldots m)$$
(4) Calculation of information entropy:
  (5) $$ej=-k\sum_{\theta =1}^{r}\sum_{i=1}^{n}p\theta ij\ln p\theta ij(k>0,k=1/\ln(rn),0\leq ej\leq 1)$$
(5) Calculation of information utility value:
  (6) gj=1−ej
(6) Calculation of index weight:
  (7) $$wj=gj/\sum_{j=1}^{m}gj(1\leq j\leq m)$$

#### 3.3.2. State Space Method

The state space method regards the evaluation object as a multidimensional space that is composed of three-dimensional state space axes of each element of the system. This paper constructs an evaluation model of tourism urbanization of major tourist cities in China by using the state space method [49,50]. The indexes of the tourism industry, urbanization, and the ecological environment are calculated. Then, the tourism urbanization index is calculated synthetically. The calculation formula is:

(8) $$Si=\sqrt{\sum_{j=1}^{n}{\left(WjXj\right)}^{2}}(i=1,2,3)$$

where Si (*i* = 1, 2, 3) are the tourism industry index, urbanization index and ecological environment index, Wj are index weights, and Xj are the spatial coordinate values of each index in the subsystem under the ideal state.

(9) $$\mathrm{TU}=|M|=\sqrt{\sum_{i=1}^{3}\sum_{j=1}^{n}{\left(WijXij\right)}^{2}}$$

where TU is the tourism urbanization index and |M| is the module of the spatial vector of tourism urbanization.

#### 3.3.3. Standard Deviation Ellipse

The standard deviation ellipse is a method for analyzing a group of points or regional trends. Its elements include the center point, long half axis, short half axis, and azimuth angle [32,48]. The center point represents the central position of all data, the long half axis represents the direction of the data distribution, and the short half axis represents the range of the data distribution. The greater the deviation of the data between the long and short half axes, the more obvious the direction of the data. Conversely, if the two are closer, the direction is less obvious. The azimuth represents the main trend of the data.

#### 3.3.4. Spatial Autocorrelation

Spatial autocorrelation is mainly used to measure the spatial correlation of geographical elements, generally including global and local spatial autocorrelation [51]. This paper uses the global Moran’s I index to explore the global correlation characteristics of tourism urbanization in China’s major tourist cities. The local correlation of tourism urbanization in 35 major tourist cities is further analyzed by using the local LISA index and Moran scatter plot.

Moran’s I is selected as the global indicator to show the global spatial distribution features of the region. The value range of Moran’s I is [−1, 1]. When Moran’s I is greater than 0, there is a positive spatial correlation. The closer the value is to 1, the more obvious the agglomeration in the spatial distribution of regions with higher or lower development levels of tourism urbanization. In contrast, when the value is closer to −1, it indicates that the spatial difference in the development level of tourism urbanization between the city and the surrounding cities is large. When Moran’s I is equal to 0, it means that there is no spatial autocorrelation [52].

Global autocorrelation reflects whether a certain observation value has agglomeration characteristics in terms of spatial distribution. It is easy to ignore some instability factors in local space. Therefore, it is necessary to test the spatial correlation of some regions by the local spatial autocorrelation method to reveal the spatial features of the development level of tourism urbanization and the degree of correlation among adjacent cities in China.

#### 3.3.5. Panel Data Model

Combined with various indicators of major tourist cities, this paper finally determined the tourism urbanization index (T) as the dependent variable. The number of A-level scenic spots (J), GDP per capita (G), urban per capita disposable income (R), end-of-year mileage per capita (K), comprehensive utilization rate of industrial solid waste (F), and green coverage rate in built-up areas (L) are independent variables [34]. The number of A-level scenic spots represents the endowment of tourism resources. GDP per capita and urban per capita disposable income represent the level of economic development. End-of-year mileage per capita represents traffic conditions. The comprehensive utilization rate of industrial solid waste and the green coverage rate in built-up areas represent policies and regional development strategies [31]. To analyze the relationship between the tourism urbanization index and various influencing factors, the panel data model is constructed as follows:

(10) Tit=αi+βiJit+δiGit+εiRit+γiKit+ηiFit+μiLit+ωit

where *i* is the province, *t* is the year, αi is the intercept term of each province, βi,δi,εi,γi,ηi,μi are the coefficients of each variable, and ωit is the random error term.

## 4. Results

### 4.1. Temporal Characteristics of Tourism Urbanization in China’s Major Tourist Cities

#### 4.1.1. Temporal Variation Characteristics of the Tourism Industry Index

**Figure 2:**
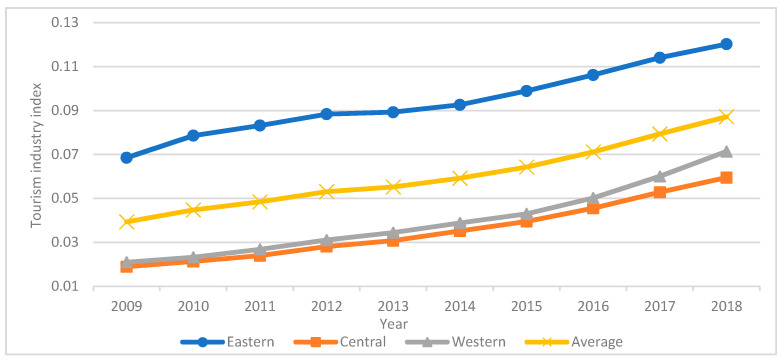
Changes in the tourism industry index in the eastern, central and western regions over time.

The state space method was used to calculate the tourism industry index of China’s major tourist cities from 2009 to 2018. Figure 2 demonstrates that the tourism industry index has risen over the last decade, with an average value of 0.0602. The eastern region has the highest tourism industry index, rising from 0.0685 in 2009 to 0.1202 in 2018, which is above average. The western region is second only to the eastern region in terms of the tourism industry index, rising from 0.0210 in 2009 to 0.0714 in 2018, which represents the largest growth but is below average. The central region has the lowest tourism industry index, rising from 0.0189 in 2009 to 0.0595 in 2018. Since 2009, Chinese people’s income and consumption levels have steadily increased. Residents’ desire for tourism consumption is increasing as the national holiday system has improved and a series of supporting measures have been implemented.

#### 4.1.2. Temporal Variation Characteristics of the Urbanization Index

**Figure 3:**
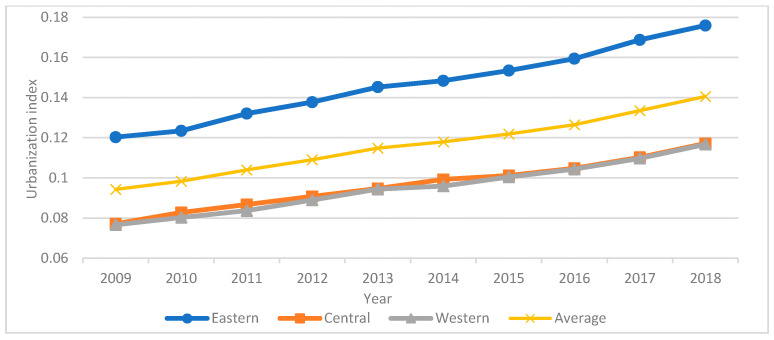
Changes in the urbanization index in the eastern, central and western regions over time.

The state space method was used to calculate the urbanization index of major tourist cities in China from 2009 to 2018. As demonstrated in Figure 3, the urbanization index has risen during the last decade, with an average value of 0.1161. The urbanization index of eastern cities is higher than average, increasing by 0.0556 between 2009 and 2018. The overall urbanization index of the central and western cities is below average. From 2009 to 2018, the central region’s urbanization index climbed by 0.04. From 2009 to 2018, the urbanization index of the western region increased by 0.0401. Since 2009, China’s urbanization has developed at a rapid pace. Furthermore, the scale of cities is continuously expanding.

#### 4.1.3. Temporal Variation Characteristics of the Ecological Environment Index

**Figure 4:**
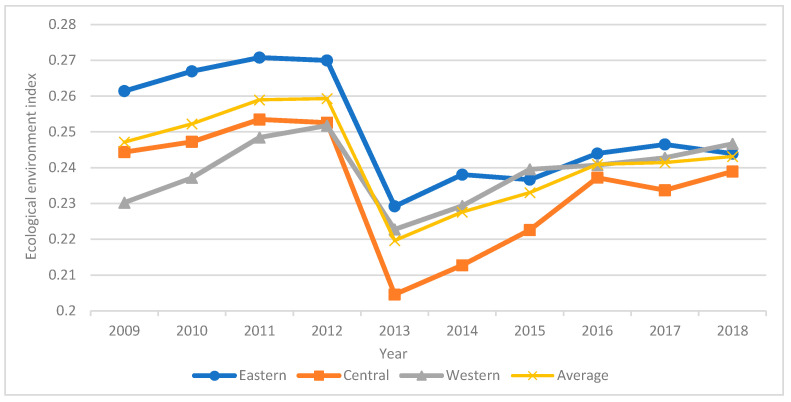
Changes in the ecological environment index in the eastern, central, and western regions over time.

The ecological environment index of China’s major tourist cities from 2009 to 2018 was calculated using the state space approach. As shown in Figure 4, compared with the tourism industry index and urbanization index, the ecological environment index fluctuated greatly, and the overall level is higher. The average value of ecological environment index was 0.2423. The ecological environment index in the eastern region has always been higher than the average level. The ecological environment index in the western region surpassed the average after 2013. The ecological environment index in the central region was below average. From 2009 to 2011, the overall ecological environment was in a stage of sustained growth. From 2012 to 2015, the ecological environmental index showed a declining and then increasing trend. In 2013, serious haze weather occurred in China, and the ecological environment gradually deteriorated. The government introduced relevant policies to adjust the industrial structure. In 2014, China began to implement the “Air pollution prevention action plan”. From 2016 to 2018, the eastern region showed a rising and then dropping trend. The western region experienced consistent growth. The central region showed a declining and then increasing trend. This demonstrates that the ecological environment has a periodicity that is tied to climate change, geographical factors, and government macro policies.

#### 4.1.4. Temporal Variation Characteristics of the Tourism Urbanization Index

**Figure 5:**
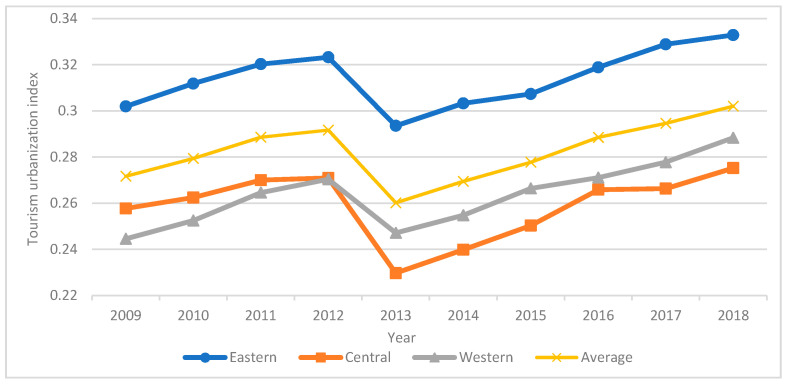
Changes in the tourism urbanization index in the eastern, central and western regions over time.

The tourism urbanization index of key tourist cities in China from 2009 to 2018 was produced using the state space approach. As indicated in Figure 5, the mean value of tourism urbanization index was 0.2824. The tourism urbanization index of the eastern region is higher than the average, while that of the central and western regions is lower than the average. From 2009 to 2011, the tourism urbanization index showed an overall upward trend with good development level. From 2012 to 2015, the tourism urbanization index showed a trend of decreasing and then increasing. In addition, the tourism urbanization index in the western region exceeded that in the central region after 2012. From 2016 to 2018, the tourism urbanization in the eastern and western regions was in a stage of rapid development, while that in the central region rose steadily. Compared with Figure 2, Figure 3, Figure 4 and Figure 5, we can find that the change trends of the tourism urbanization index and the ecological environment index are comparable before 2013. It indicates that the development of tourism urbanization is largely vulnerable to the impact of the ecological environment. After 2013, the trend is similar to that of tourism industry index and urbanization index, indicating that the impact of ecological environment is weakening and the impact of tourism industry and urbanization is strengthening. Therefore, to achieve the sustainability of tourism urbanization, we must address the mutual effect among the ecological environment, the tourism industry, and urbanization.

### 4.2. Spatial Characteristics of Tourism Urbanization in Major Tourist Cities of China

#### 4.2.1. Evolution Characteristics of the Spatial Distribution

**Figure 6:**
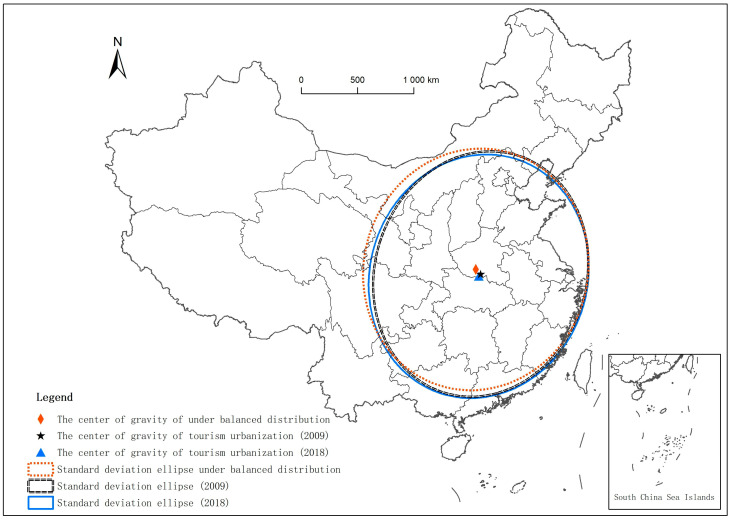
Standard deviation ellipse of tourism urbanization in major tourist cities in China.

The comparison of standard deviation ellipses between 2009 and 2018 and the equilibrium distribution is undertaken using ArcGIS software (Figure 6). When the two ellipses are closer, the distribution of cities becomes more balanced. In contrast, the disparity between cities grows. From the perspective of the center of gravity, the center of gravity of tourism urbanization under balanced conditions is roughly located in Nanyang City, Henan Province. In 2009, there was an obvious spatial dislocation between the center of gravity of tourism urbanization in reality and that under the equilibrium condition. The center of gravity shifted to the southeast of Nanyang City, Henan Province. The center of gravity of tourism urbanization in 2018 shifted slightly to the southwest relative to 2009. It was also located in the southeast direction of the center of gravity of tourism urbanization under the balanced distribution, indicating that the later change was relatively stable. From the distribution position, the standard deviation ellipse of tourism urbanization in 2009 was more inclined to the southeast than that under the equilibrium distribution, indicating that the tourism urbanization level in the southeast was better. The standard deviation ellipse of tourism urbanization in 2018 shifted to the southwest relative to 2009, and gradually approached the standard deviation ellipse under balanced distribution, indicating that the late development of tourism urbanization spread to the central and southern regions. In terms of the distribution direction, the long axis direction of the standard deviation ellipse in 2009 and 2018 was basically consistent with that under the equilibrium condition, showing a trend of “northeast-southwest”. From the length analysis of the long axis, the long axis of the standard deviation ellipse in each year was longer than that under the equilibrium condition. The short axis of each year was shorter than that under the equilibrium condition. It was the shortest and relatively sharp in 2009, indicating that the development of tourism urbanization had a certain degree of spatial agglomeration. From the standpoint of the azimuth angle change, the azimuth angle progressively grew over time, showing that cities with better tourism urbanization have a trend of expanding to the southeast.

#### 4.2.2. Global Spatial Characteristics

ArcGIS software was used to calculate Moran’s I index of tourism urbanization of major tourist cities in China from 2009 to 2018 (Table 2). The results showed that the global Moran’s I index of the tourism urbanization index was positive and passed the significance test (*p* < 0.01, Z > 2.58). This demonstrates that there is a positive spatial correlation between the development level of tourism urbanization in major tourist cities in China from 2009 to 2018; that is, cities with a higher or lower tourism urbanization index have certain agglomeration characteristics in geographical space. Moran’s I index, on the other hand, indicates a tendency of overall drop and slow recovery over time, showing that the spatial agglomeration degree of the tourism urbanization development level in China’s key tourist cities has declined over the last decade.

#### 4.2.3. Local Spatial Features

**Figure 7:**
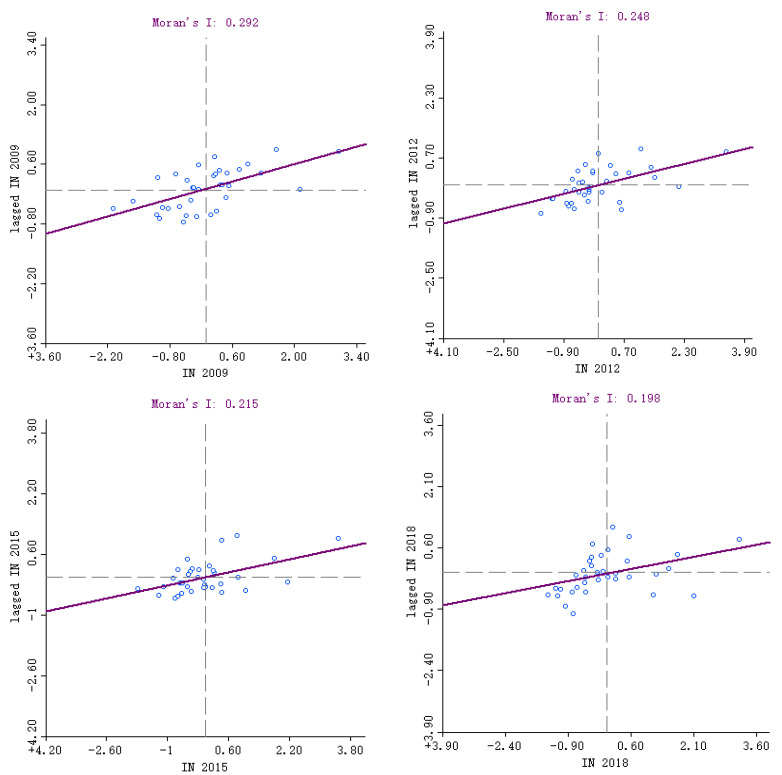
Moran scatter diagram of local autocorrelation of the tourism urbanization indexes of major tourist cities in China.

The global Moran’s I index can reflect the correlation of the tourism urbanization index in the overall spatial distribution but cannot judge the spatial correlation pattern of local areas. The Moran scatter plot of tourism urbanization indexes in 2009, 2012, 2015, and 2018 is obtained by using GeoDa software to further analyze the characteristics of local spatial agglomeration (Figure 7). As shown in Table 3, the local spatial autocorrelation types of the tourism urbanization index from 2009 to 2018 include both H-H and L-L types, which are positively correlated, and L-H and H-L types, which are negatively correlated.

(1) The first quadrant is H-H (high-value aggregation type), which means that the tourism urbanization indexes of these cities and their neighboring cities are high, and there is spatial correlation between regions. In 2009, this type was concentrated in 13 cities in Beijing-Tianjin-Hebei, the Yangtze River Delta, and the Pearl River Delta. These cities drive tourism urbanization growth with their economic development advantages. Cities of this type were primarily found in the Yangtze River Delta and the Pearl River Delta in 2012, 2015, and 2018. Suzhou, Shanghai, Hangzhou, Guangzhou, Shenzhen, Xiamen, and Sanya are among the cities that reflect this aggregation type.
(2) The second quadrant is L-H (low-high aggregation type); that is, the tourism urbanization index of the city itself is low, while the tourism urbanization level of the surrounding cities is high, and there are significant differences between regions. The cities of this type were distributed in the Beijing-Tianjin-Hebei, Pearl River Delta, Yangtze River Delta, and Chengyu Urban Agglomerations from 2009 to 2018, mainly including Tianjin, Ningbo, Guilin, Kunming, Nanchang, and other cities. These cities’ development levels are somewhat backward, the transmission effect between areas is poor, and high-quality resources from neighboring cities are not properly absorbed or utilized.
(3) The third quadrant is L-L (low-value aggregation type); that is, the tourism urbanization index of cities and adjacent cities is low, and there is spatial correlation between regions. These cities are the greatest in number, and they are concentrated in the central and western regions. Cities in these low-value agglomeration zones should seek breakthroughs in their own growth, identify urban features, achieve mutual promotion between neighboring cities, and play an active role in linking by relying on the surrounding urban agglomerations.
(4) The fourth quadrant is H-L (high-low aggregation type), which means that the city’s tourism urbanization index is high while the surrounding cities’ tourism urbanization index is low, with considerable disparities between areas. In 2009, Lijiang, Huangshan, and Yinchuan were the three major cities representing this type. Over the past decade, the number of cities of this type has increased, and Beijing, Shanghai, Chongqing, and other cities have gradually joined. Most of these cities are economically developed areas and present a unique “siphon effect”. They have obvious agglomeration advantages in all aspects of tourism urbanization development, forming a typical “core-periphery” spatial pattern and showing a certain polarization phenomenon.

**Figure 8:**
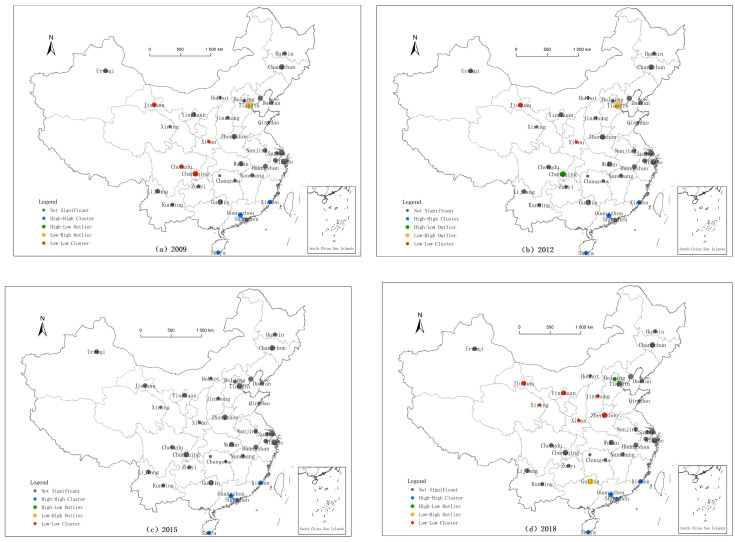
Local autocorrelation LISA clustering of the tourism urbanization indexes of major tourist cities in China.

Table 3 reveals that the H-H type cities are mostly concentrated along the eastern coast, the L-L type cities are mainly distributed in the western region, and the H-L type and L-H type cities are dispersed. Then, this distribution is verified by the LISA clustering diagram. The LISA clustering diagrams of tourism urbanization indexes in 2009, 2012, 2015, and 2018 are produced by GeoDa software. Figure 8 depicts a greater number of cities where the tourism urbanization index fails the significance test. There were four local spatial correlation models of tourism urbanization in 2009, 2012, and 2018, while in 2015, there was only a high-value aggregation model. In general, in the local spatial correlation model, the tourism urbanization of the main tourist cities in China is related and supplemented by differences. The correlation model primarily exhibits low-value clustering. This demonstrates that the level of growth of tourism urbanization in China’s key tourist cities is improving, although the effect is not noticeable.

### 4.3. Influencing Factors of the Spatiotemporal Differentiation of Tourism Urbanization in Major Tourist Cities in China

Many factors contribute to the development of tourism urbanization. According to relevant research findings [31,34], the primary factors are tourism resources, economic development level, traffic conditions, national policies, and regional development strategies. A panel data model was used to investigate the link between the tourism urbanization index and its contributing elements.

#### 4.3.1. Model Checking

This paper uses Eviews10.0 software to test the model, and the results are as follows.

According to the Hausman test (Table 4), the statistical value was 27.000958, and the *p* value was 0.0001. That is, to accept the alternative hypothesis, the fixed effect should be selected, as indicated by the likelihood ratio test (Table 5). The value of the statistics is 60.628370, and the *p* value is 0.0000, which means that the alternative hypothesis can be accepted, and the fixed effect should be selected. Based on the above two methods, the fixed effect regression model is finally chosen.

#### 4.3.2. Results and Analysis

Because of the disparities in economic development levels between cities, logarithmic processing of the model and cross-section weights can effectively solve heteroscedasticity among panel data. Table 6 displays the results (due to space constraints, the intercept term of each province is omitted here).

As shown in Table 6, the determination coefficient R^2^ is 0.922012, and the adjustment R^2^ is 0.911917, suggesting that the model fits the data very well and that the overall linear relationship is strong. The test value F is 91.32909, which is also significant. The test results and analysis of each explanatory variable are as follows:

(1) Tourism resources are the foundation of tourism development. The *p* value of the number of A-level scenic spots was less than 0.05. Hence, the original hypothesis should be rejected. It is considered that the number of A-level scenic spots has a significant impact on the tourism urbanization index, with a coefficient of 0.059313. There is a positive correlation between the two. It shows that the tourism urbanization index will increase by 5.93% when the number of A-level scenic spots increases by one unit under the condition of other variables unchanged. From 2009 to 2018, the number of A-level scenic spots increased in most areas. As of 2018, there were 254 A-level scenic spots in Beijing, 113 A-level scenic spots in Shanghai, and 239 A-level scenic spots in Chongqing. The number of scenic spots in the central and western regions is much lower than that in the eastern region.
(2) The level of economic development is the direct driver of tourism demand. The *p* values of GDP per capita and urban per capita disposable income are greater than 0.05, indicating that their influence on the tourism urbanization index is not significant enough. The coefficient of GDP per capita is 0.186238. Thus, with other factors unchanged, the tourism urbanization index will increase by 18.62% when the GDP per capita increases by one unit. The coefficient of urban per capita disposable income is 0.00960, which has little influence on the tourism urbanization index. The improvement in the economic development level of each city will support the overall development planning of the region, the upgrading of tourism products and the adjustment of the industrial structure. Since 2009, the per capita GDP of Shenzhen has been relatively high. The tourism urbanization index is as high as 0.4730. The per capita GDP of Lijiang is relatively low, and the tourism urbanization index is approximately 0.3260. The eastern regions have a higher per capita GDP and greater economic potential than the western regions. Therefore, the tourism urbanization index in the eastern region is higher than that in the western region.
(3) Traffic conditions are the basic guarantee of tourism urbanization. The *p* value of end-of-year mileage per capita is greater than 0.05, indicating that it has no significant impact on the tourism urbanization index. This indicates that highway transportation places pressure on the ecological environment, thus hindering the process of tourism urbanization. The role of this variable is not well reflected, which may be due to the lack of control variables or explanatory variables in the panel data model. In 2018, Beijing’s highway mileage reached 22,256 km, Zhangjiajie’s highway mileage increased to 9033 km, and Urumqi’s highway mileage was only 2905 km. The traffic conditions in the eastern, central, and western regions differ significantly. The land is rather flat, and the traffic network is more developed in the eastern part because the area is primarily plains. The transportation situation in the central and western is not convenient due to geography, which is not conducive to the development of tourism urbanization.
(4) National policies and regional development strategies play a guiding role in the process of tourism urbanization. The *p* value of the comprehensive utilization rate of industrial solid waste is less than 0.05, which is considered to have a significant impact on the tourism urbanization index. The coefficient is 0.026955. Accordingly, with the same other factors, the tourism urbanization index will increase by 2.69% for each unit increase in the comprehensive utilization rate of industrial solid waste. The green coverage rate in built-up areas also has a significant impact on the tourism urbanization index. The coefficient is 0.131176, indicating a positive correlation between the two. This shows that, when other factors are the same, the tourism urbanization index will increase by 13.12% if the green coverage rate in built-up areas increases by one unit. From 2009 to 2018, the green coverage rate in built-up areas decreased from 42.95% to 39.2% in Shanghai and from 39.14% to 35.37% in Harbin, while it increased from 34.27% to 41.9% in Urumqi. It is evident that the ecological environment in the eastern and central regions has been damaged to some extent, whereas the ecological environment in the western region is progressively recovering.

## 5. Discussion

By constructing a tourism urbanization indicator system, this paper discusses the spatial–temporal differentiation characteristics and influencing factors of tourism urbanization in major tourist cities in China from 2009 to 2018. The results show that, from 2009 to 2018, the tourism industry index and urbanization index of 35 major tourist cities in China increased. The fluctuation range of the ecological environment index is relatively large. The tourism industry and urbanization development are synergistic. This is consistent with the research conclusions of Xiong et al. [7] and Han et al. [34]. The tourism urbanization index was greatly affected by the ecological environment from 2009 to 2013, after which the impact of the ecological environment weakened. The impact of urbanization and the tourism industry gradually increased. Overall, the ecological environment has the strongest impact on tourism urbanization. However, in recent years, its influence has gradually declined. This confirms the interactive coupling relationship of the three subsystems. It is theoretically significant to reveal the development trend and evolution mechanism among the three subsystems. In the past decade, the development of tourism urbanization in China’s major tourist cities has presented spatial agglomeration characteristics. The local spatial correlation model is dominated by high-value agglomeration and low-value agglomeration. The H-H type cities are mainly distributed in developed eastern regions. The L-L type cities are generally distributed in the central and western regions. This result is similar to the research results of Wang et al. [13] and Ma et al. [30], indicating that the differences between the east and west have become the main characteristics of the development level of tourism urbanization in China’s major tourist cities.

This research also examines the factors that influence tourist urbanization, such as tourism resource endowment, economic development level, traffic conditions, national policy and regional development strategy. Previous scholars mostly undertook empirical studies, lacking a theoretical basis [33,34]. This paper constructs a panel data model for testing and further discusses the intensity of each influencing factor on tourism urbanization. This shows that the guiding role of national policies and regional development strategies is the strongest. This is similar to the research results of Che et al. [10] and is basically consistent with China’s national conditions. Second, tourism resources and economic development level have a strong effect on tourism urbanization. The higher the abundance of tourism resources in a region, the more likely that the development of tourism urbanization will be promoted. The higher the level of economic development, the more people’s consumption demand will be stimulated, thus driving the development of tourism urbanization. Finally, Gao et al. [11] and Xiong et al. [7] believe that the higher the traffic accessibility, the more concentrated the tourism industry will be, and the greater the impact on tourism urbanization. However, this paper contends that the construction of roads damages the ecological environment to some extent. Therefore, it is necessary to establish a reasonable traffic network layout, support its leading role, and promote economic development throughout the network.

## 6. Conclusions

The coordinated development of the tourism industry, urbanization, and the ecological environment is of great significance for the sustainable development of tourist cities, and the three interact and influence each other. Based on the panel data of 35 major tourist cities in China from 2009 to 2018, this paper analyses the spatial and temporal differentiation characteristics and influencing factors by using the state space method, standard deviation ellipse, and spatial autocorrelation analysis. The research results further enrich the dimension and depth of tourism urbanization research, and provide reference for local governments to formulate coordinated development strategies.

During the research period, the development level of the tourism industry index is the lowest, the urbanization index development level is relatively high, and both indexes are increasing. The ecological environment index presents the highest development level, and the fluctuation range is relatively large. The tourism urbanization index indicates a fluctuating growing trend under the combined influence of the three elements. The development center of tourism urbanization shifts southeastward, and the distribution direction is northeast-southwest. There is a certain spatial agglomeration, but it weakens over time. The local spatial correlation pattern is dominated by correlation characteristics and supplemented by different characteristics. The eastern region is dominated by high-value aggregation cities, whereas the middle and western regions are dominated by low-value aggregation cities. Many factors influence tourism urbanization. Overall, national policies and regional development strategies are the most important, followed by tourism resource endowment, economic development levels, and traffic conditions.

Based on the study of spatial–temporal differentiation characteristics and influencing factors of tourism urbanization in China’s major tourist cities, we put forward some practical suggestions:

(1) Clear the direction of tourism urbanization. The development levels of China’s tourism industry and urbanization must be improved. We should increase investment in tourism funds and improve basic supporting facilities. Strengthening the ecological civilization construction of tourism cities lays the foundation for the high-level development of tourism urbanization. Promote the coordinated development of the tourism industry and urbanization on the basis of protecting the ecological environment. Areas with a high level of tourism urbanization should continue to maintain advantages and build influential tourism cities. Regions with a low level of tourism urbanization should extend the industrial chain, optimize the industrial structure and reduce pollution.
(2) Narrow the development gap between eastern and western tourism urbanization. Based on the actual development situation, each city should give full play to its own advantages and formulate differentiated development strategies for different target groups. We should expand exchanges and cooperation among cities, strengthen the complementarity of resource advantages, improve market competitiveness, and accelerate the urbanization process. The eastern region should grasp its advantages in economic development and give full play to its radiating and driving role in stimulating the economic development of the surrounding areas. The central and western regions should rationally allocate resources to cultivate new “high-value cluster areas” and narrow the regional gap.
(3) Strengthening the positive influence of driving factors on tourism urbanization. The government should increase the support of financial and technical personnel, train and introduce excellent talent, and improve the quality and level of tourism services. Appropriate adjustments should be made to tourism development planning and protection based on regional resource characteristics and ecological advantages. A sound transportation network system should be established to improve accessibility between cities. In the process of tourism urbanization development, we should adhere to scientific, rational, and orderly development, and improve the quality of the ecological environment.

This paper still has certain limitations. First, the construction of the evaluation index system of tourism urbanization is not comprehensive enough. Different scholars have sought to select indicators from various angles. The index system of tourism urbanization is not unified at present. The selection of indicators may lead to deviation of the results, and objectivity needs to be verified. Second, tourism urbanization is influenced by a variety of causes, and the dominating factors vary among places and time periods. Further investigation into the driving mechanism’s changing process is possible. Third, due to the limitation of data availability, the time span (2009–2018) of this study is relatively short. Future studies may use our study as a point of departure to track changes across broader time parameters.

## Figures and Tables

**Table 1:** Evaluation index system of the tourism–urbanization–ecological environment system.

Subsystem	Evaluation Index	Unit	Nature	Weight
Tourism Industry	Number of inbound tourists	10,000 people	+	0.1501
	Domestic tourism	10,000 people	+	0.1067
	Foreign exchange earnings from tourism	USD 10,000	+	0.1667
	Domestic tourism revenue	100,000,000 yuan	+	0.1006
	Total tourism revenue	100,000,000 yuan	+	0.0998
	Tourism income as a percentage of GDP	%	+	0.1291
	Number of A-level scenic spots	a	+	0.0795
	Number of star hotels	a	+	0.1056
	Number of travel agencies	a	+	0.0619
Urbanization	Urban per-capita disposable income	yuan/person	+	0.0956
	Urban road area per capita	m^2^/person	+	0.1008
	End-of-year mileage per capita	km/person	+	0.1001
	Urban end-of-year registered unemployment rate	%	-	0.0754
	Number of public vehicles in operation	a	+	0.2788
	GDP per capita	yuan/person	+	0.1037
	Secondary industry as a percentage of GDP	%	+	0.0475
	Tertiary industry as a percentage of GDP	%	+	0.0938
	Proportion of employed population in the secondary industry	%	+	0.0533
	Proportion of employed population in the tertiary industry	%	+	0.0510
Ecological Environment	Industrial sulfur dioxide emissions	10,000 tons	-	0.0517
	Industrial soot emissions	10,000 tons	-	0.0100
	Industrial wastewater discharge	10,000 tons	-	0.0841
	Green coverage rate in built-up areas	%	+	0.1372
	Per capita park green area	hm^2^/person	+	0.2396
	Days of air quality at level 2 or higher	a	+	0.2031
	Comprehensive utilization rate of industrial solid waste	%	+	0.1375
	Centralized treatment rate of urban sewage	%	+	0.0693
	Pollution-free treatment rate of domestic garbage	%	+	0.0675

**Table 2:** Global Moran’s I value and test results of the tourism urbanization index of major tourist cities in China.

Year	Moran’s I Index	Z Score	*p* Value
2009	0.202521	2.870520	0.004098
2010	0.227301	3.186548	0.001440
2011	0.220067	3.114921	0.001840
2012	0.197731	2.867791	0.004133
2013	0.199862	2.852398	0.004339
2014	0.163579	2.403359	0.016245
2015	0.193124	2.842416	0.004477
2016	0.187318	2.727239	0.006387
2017	0.214422	3.057489	0.002232
2018	0.225018	3.162057	0.001567

**Table 3:** The types of local spatial autocorrelation of the tourism urbanization indexes of major tourist cities in China.

Year	H-H	L-H	L-L	H-L
2009	Beijing, Suzhou, Shanghai, Hangzhou, Guangzhou, Shenzhen, Xiamen, Sanya, Qinhuangdao, Dalian, Qingdao, Kunming, Hohhot, Changchun (14)	Ningbo, Guilin, Jinzhong, Nanchang, Nanjing, Tianjin and Harbin (7)	Zunyi, Zhangjiajie, Jiuquan, Chongqing, Chengdu, Changsha, Wuhan, Xi‘an, Zhengzhou, Xining, Urumqi (11)	Lijiang, Huangshan, Yinchuan (3)
2012	Suzhou, Shanghai, Hangzhou, Guangzhou, Shenzhen, Xiamen, Sanya, Qinhuangdao (8)	Ningbo, Guilin, Dalian, Kunming, Nanchang, Tianjin, Changchun, Xiamen(8)	Zunyi City, Zhangjiajie City, Jiuquan City, Jinzhong City, Chengdu City, Changsha City, Wuhan City, Xi’an, Zhengzhou, Nanjing, Xining, Yinchuan, Urumqi, Hohhot, Harbin (15)	Lijiang, Huangshan, Chongqing and Beijing (4)
2015	Suzhou, Shanghai, Hangzhou, Guangzhou, Shenzhen, Xiamen, Sanya (7)	Ningbo, Guilin, Dalian, Kunming, Nanchang, Tianjin, Changchun, Chengdu (8)	Zunyi, Zhangjiajie, Jiuquan, Jinzhong, Changsha, Wuhan, Zhengzhou, Nanjing, Xining, Xi’an, Yinchuan, Urumqi, Hohhot and Harbin (14)	Lijiang, Huangshan Qingdao, Chongqing, Shanghai and Beijing (6)
2018	Suzhou, Shanghai, Hangzhou, Guangzhou, Shenzhen, Xiamen, Sanya (7)	Ningbo, Guilin, Kunming, Nanchang, Changchun, Qinhuangdao, Chengdu, Changsha (8)	Zhangjiajie, Jiuquan, Jinzhong, Dalian, Wuhan, Xi‘an, Zhengzhou, Xining, Yinchuan, Tianjin, Urumqi, Hohhot, Harbin (13)	Lijiang, Zunyi, Huangshan, Chongqing, Qingdao, Nanjing and Beijing (7)

**Table 4:** The results of the Hausman test.

Effects Test	Statistic	Chi-Sq.d.f.	Prob.
Cross-section random	27.000958	6	0.0001

**Table 5:** The results of likelihood ratio test.

Effects Test	Statistic	d.f.	Prob.
Cross-section F	60.628370	(34,309)	0.0000

**Table 6:** The results of model estimation.

Variable	Coefficient	Std.Error	t-Statistic	Prob
ln C	−2.453413	0.259174	−9.466280	0.0000
ln J	0.059313	0.019613	3.024223	0.0027
ln G	0.054267	0.030994	1.750868	0.0810
ln R	0.009660	0.032415	0.298026	0.7659
ln K	−0.037548	0.032006	−1.173139	0.2416
ln F	0.026955	0.013670	1.971902	0.0495
ln L	0.131176	0.019555	6.708153	0.0000
**Weighted Statistics**				
R-squared	0.922012		F-statistic	91.32909
Adjusted-R-squared	0.911917		Prob (F-statistic)	0.000000

## Data Availability

Data is contained within the article.
